# Innate Immune System in the Pathogenesis of Non-Alcoholic Fatty Liver Disease

**DOI:** 10.3390/nu15092068

**Published:** 2023-04-25

**Authors:** Dae Won Ma, Joohun Ha, Kyung Sik Yoon, Insug Kang, Tae Gyu Choi, Sung Soo Kim

**Affiliations:** 1 Department of Biochemistry and Molecular Biology, School of Medicine, Kyung Hee University, Seoul 02447, Republic of Korea; truelight1209@hotmail.com (D.W.M.); hajh@khu.ac.kr (J.H.); sky9999@khu.ac.kr (K.S.Y.); chtag@khu.ac.kr (T.G.C.); sgskim@khu.ac.kr (S.S.K.); 2 Department of Medicine, Graduate School, Kyung Hee University, Seoul 02447, Republic of Korea; 3 Biomedical Science Institute, Kyung Hee University, Seoul 02447, Republic of Korea

**Keywords:** non-alcoholic fatty liver disease, hepatic inflammation, sterile inflammation, damage-associated molecular patterns, pattern recognition receptors, NLRP3

## Abstract

Non-alcoholic fatty liver disease (NAFLD) is a prevalent condition characterized by lipid accumulation in hepatocytes with low alcohol consumption. The development of sterile inflammation, which occurs in response to a range of cellular stressors or injuries, has been identified as a major contributor to the pathogenesis of NAFLD. Recent studies of the pathogenesis of NAFLD reported the newly developed roles of damage-associated molecular patterns (DAMPs). These molecules activate pattern recognition receptors (PRRs), which are placed in the infiltrated neutrophils, dendritic cells, monocytes, or Kupffer cells. DAMPs cause the activation of PRRs, which triggers a number of immunological responses, including the generation of cytokines that promote inflammation and the localization of immune cells to the site of the damage. This review provides a comprehensive overview of the impact of DAMPs and PRRs on the development of NAFLD.

## 1. Introduction

The global rise in the incidence of diabetes mellitus, obesity, and metabolic syndrome has led to a corresponding increase in the prevalence of non-alcoholic fatty liver disease (NAFLD), which is estimated to affect one-quarter of the global population [1]. Nonalcoholic steatohepatitis, a severe form of NAFLD characterized by increased hepatic inflammation, fibrosis, and mortality, affects up to 30% of all NAFLD patients [2]. Additionally, it can lead to liver cirrhosis, making it the most common indication for liver transplantation in both Europe and the USA [1,3].

Hepatic inflammation can result in various metabolic liver diseases [4]. The conventional understanding of inflammation is limited to the identification of foreign pathogens by the innate immune system to combat infection. However, this concept fails to account for inflammation that is independent of the microbiome. Recent research suggests that the innate immune system can respond to endogenous antigens in addition to exogenous pathogens [5]. Inflammation triggered by the recognition of self-antigens by the innate immune system is referred to as sterile inflammation (SI).

The study of SI has made significant progress in comprehending how tissue injury leads to inflammation. This theoretical framework originated from the limitations of self-awareness and non-self-awareness in demonstrating the specificity of the adaptive immune system. Consequently, it was proposed that the presence of danger signals is a prerequisite for immunologic activation, and that molecules called damage-associated molecular patterns (DAMPs), which are generated from injured cells, act as molecular stimulants for inflammatory responses [5,6,7]. Recent research has identified several DAMPs, their receptors, and the cellular mechanisms that orchestrate full-blown inflammation. Recently, numerous DAMPs have been found to be involved in hepatic injury and NAFLD. Therefore, it is essential to understand how DAMPs contribute to the development of SI in NAFLD.

Pattern recognition receptors (PRRs) alert immune cells to cellular damage by identifying DAMPs [8]. Pro-inflammatory mediators are upregulated by this mechanism, which enables the immune system to respond to both infectious and non-infectious threats to the tissue [9]. Purinergic P2X7 receptors, toll-like receptors (TLRs), GMP-AMP synthase (cGAS), and the NLR family pyrin domain containing 3 (NLRP3)-inflammasome are key players in the connection between cellular injury and the subsequent inflammatory response [10].

## 2. NAFLD Pathogenesis

The precise process for the development of NAFLD is not yet clear. Insulin resistance appears to play a crucial role in the development of the NAFLD [11]. Genetic mutations in PNPLA3, an enzyme that encodes I148M and is involved in the hydrolysis of triacylglycerols in adipocytes, have been linked to NAFLD in the absence of metabolic syndrome [12,13]. Additionally, TM6SF2, a genetic variant of a lipid transporter placed in the endoplasmic reticulum, leads to the dysfunction of proteins and increases the hepatic accumulation of triglycerides [14]. The pathological aggravation of NAFLD is as follows: steatosis, lipotoxicity, and inflammation [15].

The interaction of nutrition, genetic factors, gut microbiota, and de novo lipogenesis causes steatosis by upregulation of lipogenic transcription factors [12,16,17,18]. Fatty acids are kept as triacylglycerols in the adipose tissue, but in obese patients, fatty acids appear to be misdirected from their main storage location to ectopic areas, including skeletal or hepatic tissue, where they are re-esterified to diacylglycerol. The absorption of fatty acids is probably promoted by fatty acid transport proteins and translocases, which have been known to be increased in patients with NAFLD [19,20]. Steatosis causes the up-regulation of the transcription factor NF-κβ (nuclear factor—kappaβ). The activation of NF-κβ drives the production of pro-inflammatory mediators, such as tumor necrosis factor-alpha (TNF-α), IL-6, and IL-1β [21]. They promote the gathering and activation of Kupffer cells (KCs) to mediate inflammation in the NAFLD [18,22].

Excessive lipids in the liver induce lipotoxicity and result in the loss of function of organs, primarily resulting in mitochondrial failure and endoplasmic reticulum stress [23]. Dysfunctional mitochondria have a heightened ability to oxidize fatty acids, generating reactive oxygen species; an imbalance between reactive oxygen species and protective oxidant production results in oxidative stress [24]. Oxidative stress in NAFLD patients is regarded as the third offense, which eventually causes hepatocyte death [25]. As NAFLD advances, it results in intricate alterations in both the hepatic histopathology and biochemical features, thereby perpetuating a vicious cycle of disease progression.

The transcription factors known as hypoxia-inducible factors (HIFs) are crucial in the cellular response to hypoxia, or low oxygen levels [26]. In the context of NAFLD, HIFs may contribute to the onset and aggravation of the disease. HIFs are activated in response to hypoxia, and once activated, they regulate the expression of genes involved in angiogenesis, erythropoiesis, glucose metabolism, and cell survival. HIFs may contribute to the development of the disease by promoting the accumulation of fat in the liver, the production of pro-inflammatory cytokines, and the development of liver fibrosis [27].

Small non-coding RNA molecules called microRNAs (miRNAs) are essential for post-transcriptional gene control [28]. They bind to the 3′ untranslated region of target messenger RNAs, leading to translational inhibition or messenger RNA degradation. In the context of NAFLD, miRNAs have been found to be dysregulated and to play a role in the development of liver damage. Studies have identified several miRNAs that are dysregulated in NAFLD, including miR-122, miR-34a, miR-21, and miR-155 [29,30]. These miRNAs have been found to control a number of mechanisms, including lipid metabolism, inflammation, and fibrosis, that support the onset and progression of NAFLD.

### 2.1. DAMPs

The terminology “DAMPs” is widely used to refer to molecules that have the capability to initiate inflammation itself [5]. Various DAMPs have been discovered, and this number is increasing [31]. For many years, it has been recognized that the cytoplasm of apoptotic cells can trigger dendritic cells (DCs) and therefore contain DAMPs. Uric acid was identified as a DAMP by chromatographic separation, and it needs to be crystallized in order to promote SI [32]. DAMPs bind to PRRs, inducing an inflammatory response and developing an injury expansion, resulting in tissue damage (Table 1). TLRs are the best PRRs, with a family of cell surface and endocytic receptors found in most hepatic cells, including KCs, hepatic stellate cells, biliary epithelial cells, and sinusoidal endothelial cells [33,34,35]. The well-known hepatic TLRs are TLR2, TLR4, and TLR9 [34,35,36].

### 2.2. DNA and Nucleotide

A well-known TLR9 activator is bacterial CpG DNA. However, endogenous denatured DNA of the host’s apoptotic and necrotic cells can also activate the TLR9-dependent immune responses, including antibody formation, DC maturation, and the induction of inflammatory mediators [37]. TLR9 activation upregulates pro-IL-1β and pro-IL-18 genes in sinusoidal endothelial cells, which prepares these cells for caspase-1 assembly with the NLRP3 inflammasome upon stimulation by ATP for P2X7 [38]. TLR9 activation upregulates pro-IL-1 and pro-IL-18 genes in these cells TLR9 mRNA, type I interferon (IFN), and IL-1 were overexpressed in KCs/macrophages in tandem with the progression of steatohepatitis in a model of methionine- and choline-deficient diets [39]. Additionally, the liver damage, apoptosis, inflammation, steatosis, and fibrosis that are present in NAFLD are aggravated by the DNA-sensing receptor cGAS, an extra cytosolic PRR for host DNA [40,41].

Nucleotide metabolism is the process by which nucleotides (the building blocks of DNA) are synthesized and broken down in the body [42]. It has been shown that alterations in the nucleotide metabolism can contribute to the development of NAFLD. Adenosine is a purine nucleoside that is essential for controlling cellular signaling and metabolism [43]. Adenosine signaling has been demonstrated to control many physiological processes, such as inflammation, metabolism, and fibrosis, all of which are implicated in the pathophysiology of NAFLD.

Several investigations have shown that adenosine signaling is dysregulated in NAFLD [44]. NAFLD patients have higher amounts of adenosine in their livers, which is likely to have a role in the development of fibrosis, inflammation, and insulin resistance. Adenosine signaling is mediated by four G-protein-coupled receptors (A1, A2A, A2B, and A3) [45]. It has been demonstrated that activating the A1 and A3 receptors promotes hepatic inflammation while activating the A2A and A2B receptors has a protective effect against fibrosis and inflammation [46].

Taken together, these data suggest that the dysregulation of the nucleotide metabolism and adenosine signaling may promote inflammation, fibrosis, and a dysregulated lipid metabolism, which may contribute to the onset and worsening of NAFLD. For the treatment of NAFLD, targeting these pathways may constitute a potential therapeutic strategy.

### 2.3. High-Mobility Group Protein B1 (HMGB1)

HMGB1 is a non-histone nuclear protein that promotes DNA binding with regulatory proteins and generally induces their transcriptional activation [47]. It is able to be released from necrotic hepatocytes as a warning sign of liver injury [48,49]. Following exposure to microbial damage, it can also seep from hepatocytes, attracting inflammatory cells through chemoattraction [50,51]. The nature of HMGB1 is much more complicated than that of other DAMPs since it reacts with various receptors [51]. They have extra ligands, and HMGB1 appears to operate to set the magnitude of the reaction after specific receptor–ligand interactions. HMGB1 has no direct pro-inflammatory effects, but it induces inflammation by acting in combination with other pro-inflammatory mediators, such as lipopolysaccharides (LPS), single-stranded DNA, nucleosomes, and IL-1β [52]. When HMGB1 activates macrophages, TLR4 acts as a major receptor [53]. It has a lower tendency to bind with HMGB1 than LPS and has the identical nuclear factor κB pathway, but it initiates different profiles of gene expression [54]. Additionally, the signal of TLR4 can facilitate the explosion of HMGB1 through increased reactive oxygen species and the up-regulation of calcium and the transcription factor interferon regulatory factor 1 (IRF1) in the cytosol [55]. As a result, IRF1 alters its acetylation state, leading to nuclear HMGB1’s translocation to the cytosol [56].

### 2.4. ATP

Substantial quantities of ATP are present in the cytosol and can be emitted by dead cells and act as DAMPs by ligating the P2X7 receptors [57]. Plasma membrane pores are formed as a result of the interaction between ATP and the P2X7 receptor, which also causes a potassium efflux and a calcium influx [58]. Sustained cellular P2X7 receptor activation may induce solute waste and cell death [59]. This mechanism is regulated by the fast metabolism of ATP to adenosine diphosphate by CD39 and then to adenosine by CD73 [60]. The function of ATP as a DAMP in NAFLD is not clear. In overweight mice exposed to carbon tetrachloride, the P2X7-NADPH oxidase axis was responsible for recognizing oxidative stress and ATP released from hepatocytes that triggered the inflammation of Kupffer cells [61]. In contrast, mice lacking the P2X7 receptor showed a constant response in their adipose tissue compared with wild-type mice in a high-fat diet model [62].

### 2.5. Uric Acid

Crystallization occurs when uric acid is excreted from cells, and this crystallization is a crucial factor in determining the intensity of the inflammatory response [32]. It is known that the sodium concentration and protein components of the extracellular fluid contribute to the crystallization [63]. Genetic and pharmacological interventions aimed at lowering uric acid levels reduce aseptic damage in the liver [64,65].

When hepatocytes are destroyed, the nucleic acid is broken down, and uric acid is released [65]. When uric acid levels increase and reach supersaturation levels, they are transformed into crystals and join the surface lipids of immune cells, activating the NLRP3-inflammasome to induce IL-1β-mediated release [64,66]. Dysfunction of mitochondria and lipogenesis induced by fructose occur in hepatocytes affected by uric acid crystals [67]. In addition, insulin resistance, oxidative stress, and the exacerbation of NAFLD are all linked to elevated uric acid [68]. It is known that when uric acid accumulates, it activates NLRP3-inflammasome and exacerbates fat accumulation and insulin resistance in hepatocytes [69]. Additionally, it is known that xanthine oxidase, an enzyme involved in the production of uric acid, regulates the activity of the NLRP3-inflammasome in NAFLD [70].

### 2.6. Fatty Acids

Visceral adipose tissue can induce systemic inflammation through the increased production of adipocyte IL-8 and TNF-α in obese individuals [71]. Saturated palmitic acid has been shown to enhance the release of HMGB1 by hepatocytes both in vitro and in vivo, subsequently activating the TLR4/MyD88 signaling axis and leading to the overexpression of cytokines [72]. Furthermore, palmitic acid can promote the stimulation of NLRP3-inflammasomes in liver cells, which sensitizes them to LPS-induced IL-1β production [73].

### 2.7. Cholesterol

Dietary cholesterol, along with triglycerides, probably contains additional DAMPs for the rapid conversion of NAFLD to liver inflammation when it accumulates inside the liver [74]. There are two mechanisms by which cholesterol damages hepatocytes: direct damage and indirect damage. The former happens when oxysterol is produced, causing endoplasmic reticulum stress and mitochondrial oxidative damage, whereas the latter happens when adipose tissue malfunctions [75]. When consumption of cholesterol is reduced, there is an increase in its production and absorption from food, which leads to hepatic storage [76]. Although mice lacking the LDL receptor were fed a diabetic diet and showed some changes in terms of liver steatosis and fibrosis, a diabetes diet with cholesterol exacerbated liver symptoms, with increased immune cell congestion and hepatocellular oxidative insult [77]. Cholesterol may act as a DAMP to induce liver inflammation in NAFLD.

### 2.8. Mitochondria

The dysfunction of mitochondria in the liver is known to be a cause of NAFLD [78]. Injured mitochondrial DNA can evade autophagy, which can activate TLR9 [79]. Diverse warning signs beginning from mitochondria, such as formylated peptides, ATP, and ROS, can induce an SI by activating MAPKs, cGAS/STING, and NLRP3-inflammasomes [80,81].

### 2.9. Histones

It is unclear how histone proteins, which are found in DNA in the nucleus, contribute to the development of NAFLD. It is known that circulating histones increase after ischemic hepatic damage, resulting in DNA-mediated TLR9 activation [82]. In addition, extracellular histone proteins activate TLR2- and TLR4-dependent inflammatory pathways and control cell death when a sterile inflammatory response occurs [83]. Furthermore, fatty liver-associated hepatocellular cancer in humans showed increased hepatic expression of a particular histone protein [84].

### 2.10. PRRs

Recently, various studies have identified innate immune responses that contribute to the development of NAFLD. The activation of immune cells in the liver by adipose tissue- or intestinal-derived signals influences hepatocyte damage and NAFLD progression. In particular, PRR is known to have a significant role in identifying cell damage in NAFLD and appears intracellularly or on the surfaces of cells [85].

### 2.11. TLRs

TLRs are PRR proteins that are crucial for innate immunity and have the ability to detect and activate defenses against bacterial, fungal, and viral raiders. Ten different TLR types have been identified in humans and 12 different TLR types have been identified in mice, according to a recent study [86]. TLRs can detect not only the microbiome but also endogenous DAMPs. HMGB1, DNA, RNA, and S100 proteins are well-known ligands for TLRs among these DAMPs [87,88]. Additionally, they detect cells destroyed by necrosis or apoptosis as DAMPs and induce an inflammatory response in the liver [38,89].

In patients with NAFLD, an intestinal bypass develops, resulting in elevated levels of serum TLR4 ligands, including lipopolysaccharides [90]. Conversely, the absence of TLR4 and TLRs in patients with NAFLD improves fibrosis markers, ALT levels, histological scores, and hepatocyte apoptosis [39,91,92]. TLR9 is needed for IL-1β production in KC, which leads to hepatocyte apoptosis in NAFLD. According to recent research, IL-1β does not directly cause cell death; rather, it causes hepatocytes to be more receptive to pre-existing apoptotic signals [39,93].

### 2.12. P2X7 Receptor

ATP stimulates the purinergic (P2) family of receptors to transduce signals extracellularly [94]. Seven P2X and eight P2Y types of receptors have been identified so far, of which the P2X7 receptor has been studied the most [59]. When the P2X7 receptor is stimulated, potassium ions are rapidly released from the cytoplasm, signals are transmitted to the NLRP3-inflammasome in the cell, and IL-1β and 18 activations occur through the caspase-1 mediation [95,96]. The adapter molecule ASC (apoptosis-associated speck-like protein containing a carboxy-terminal caspase recruitment domain) can be ligated to create the NLRP3-inflammasome. For example, ASC-deficient mice cannot cut caspase-1 and handle IL-1β maturation after ATP binds to the P2X7 receptor [97].

### 2.13. NLRP3-Inflammasome

The caspase-1 cascades and the activation of other inflammatory responses depend on the cytoplasmic protein complex known as the inflammasome. DAMPs and crystalline and particulate matter activate the NLRP3-inflammasome [98]. The NLRP3-inflammasome’s *N*-terminal pyrin domain (PYD) interacts with the PYD domain of the ASC to cleave procaspase-1. Caspase-1 that has been cleaved transforms into active states that can be secreted, such as pro-IL-1β and 18 [99].

The generation of the NLRP3-inflammasome has been proven to occur in hepatocytes, KCs, sinusoidal endothelial cells, and hepatic stellate cells [38,73,100,101]. Since HFD-induced hepatic steatosis was lessened in knockout mice for NRP3, NLRP3-inflammasome activation may correlate with NALFD aggravation [102]. In addition, a mouse model’s incidence of steatohepatitis and fibrosis was decreased when liver parenchymal cells lacked IL-1β [103]. Meanwhile, increased assembly components in the HFD model increased the incidence of hyperglycemia and hyperinsulinemia [62]. On the other hand, mice lacking these assembly components exhibited reduced risk for obesity and insulin resistance, even when exposed to an HFD [104].

## 3. Cellular Interactions

Several cells present in liver fibrosis play an important role in the persistence of inflammation, the activation of mesenchymal cells, the accumulation of the extracellular matrix, and the resolution of fibrosis (Table 2).

### 3.1. Kupffer Cells

KCs are known as macrophages that reside in the liver and are located in the hepatic lymph nodes, hepatic sinusoids, and portal vein [105]. They are the most prevalent population of tissue-specific macrophages and are derived from circulating monocytes. They constitute approximately 15 percent of hepatic cells [106]. Pathogens or products formed from bacteria that enter the portal circulation are phagocytosed by KCs in order to stop their dissemination to the peripheral circulation. Additionally, KCs are responsible for phagocytosing cell debris from adjacent cells and presenting antigens to Furthermore, KCs are in charge of phagocytosing cell debris from neighboring cells and presenting antigens to regulatory and cytotoxic T lymphocytes. Several studies have demonstrated that reducing the number of KCs can alleviate insulin resistance, inflammation, and fibrosis. Consequently, the stimulation of KCs plays a key role in the development and exacerbation of NAFLD [107]. KCs are elevated during the early stages of NAFLD before other immune cells are enlisted [108].

Depending on the surrounding circumstances, KCs can go through various types of activation similar to other macrophages [105]. Therefore, KCs display diverse polar morphologies, which can be classified into the activated M1 macrophages phenotype and the alternative M2 phenotype. The M1 phenotype is considered to be pro-inflammatory in nature, whereas the M2 type is considered “immunomodulatory” because it is involved in recovery and anti-inflammatory, in addition to being pro-inflammatory, in several cases. This binary idea, though, may not accurately represent the biology of the various macrophage subgroups, as KCs can occasionally exhibit both the M1 and M2 markers in some special circumstances [109].

Pathogen-associated molecular patterns containing LPS and other gut-derived bacterial metabolites are the main stimulants that result in the characteristic M1 phenotype of KCs. They link to the TLRs of KC and produce pro-inflammatory cytokines and chemokines like IL-1β, IL-12, and TNF-α. This is an essential step in the release of DAMPs and works by inducing local inflammation and promoting further hepatocellular damage. DAMPs activate KCs through the TLR signaling pathway, promoting a vicious cycle. The chemotaxis of inflammatory cells and the provocation of hepatic stellate cells are both affected by the pro-inflammatory and pro-fibrotic qualities of several of the cytokines mentioned above [110]. Several studies have shown that KC may be activated by lipid overload in the context of NAFLD. This may include other lipids, such as fatty acids, ceramide diacylglycerols, oxidized lipoproteins, and free cholesterol, which have been reported to increase inside the KC of NAFLD; this determines the overexpression of TLRs and the activated reaction to LPS [111,112]. Therefore, fat overload in KCs also appears to contribute to NAFLD progression by inducing the activation of these cells.

Not only the M1 polarized style, but also KCs can adopt an alternative M2 phenotype, which involves the secretion of a unique collection of mediators such as IL-13, IL-10, IL-4, and TGF-α, and an interaction with Th2 CD4 T-cells. M2 phenotypes are mostly linked to the healing of wounds and the subsiding of inflammatory conditions [105,109]. Nonetheless, the characteristics of these polarized KCs are multifaceted, and several other subtypes have also been identified, each with distinct functional capabilities and regulatory mechanisms. Activating the nuclear receptor PPAR- δ facilitates M2 activation and improves obesity-induced glucose intolerance in mice, suggesting that this route may be used for NAFLD treatment despite the unknown function of M2 polarization of KCs in NAFLD [105,113].

### 3.2. Dendritic Cells

DCs are a population of tolerogenic immune cells that make up a small fraction of the body of hepatic non-parenchymal cells and are distributed around the central and portal veins. DCs can function as antigen-presenting cells, as well as limiting SI by acting as apoptotic cells and necrotic debris clearers. The role of DCs in NAFLD is complex and widely debated [114,115]. While immature and tolerogenic DCs are dominant under physiological conditions, mature and proinflammatory DCs are thought to dominate during liver injury. DCs quickly penetrate the liver, displaying an activated immunophenotype expressing increased levels of IL-6, TNF-α, and monocyte chemoattractant protein-1 (MCP-1) [114]. Paradoxically, the depletion of DCs in the liver leads to exacerbated liver inflammation and fibrosis, suggesting that DCs may play anti-inflammatory and anti-fibrotic roles in NAFLD. A previous study suggests that some dendritic cells (DCs) found in the inflamed liver may arise from monocytes that express both inflammatory monocyte and DC markers on their cell surface, along with fractalkine receptors [116]. This particular DC subtype exhibits an enhanced ability to produce inflammatory mediators and present antigens. Inhibiting these cells in a mouse model of NAFLD resulted in decreased levels of TNF-α and prevented hepatic damage [116].

### 3.3. Neutrophils

Neutrophil accumulation in the liver is a characteristic feature of NAFLD. This phenomenon is believed to play a crucial role in liver injury by instigating macrophage recruitment and exacerbating prior inflammation through interactions with antigen-presenting cells. The release of myeloperoxidase, a pro-oxidant enzyme produced by neutrophils, is thought to be an associated process that enhances macrophage cytotoxicity and facilitates inflammation and fibrosis in experimental models [117]. Studies have demonstrated that NAFLD development and hepatic pro-inflammatory cytokine production can be reduced in myeloperoxidase-deficient mice [118]. Neutrophil-derived peptides were also shown to promote liver fibrosis in an experimental NAFLD model [119]. Furthermore, earlier investigations confirmed that elastase, another protease derived from neutrophils, regulates hepatic insulin resistance by promoting an inflammatory response. This observation is supported by other studies that reveal the amelioration of liver inflammation through the deficiency of neutrophil elastase [120].

### 3.4. Natural Killer and Natural Killer T Cells

Natural killer (NK) cells are a subset of lymphocytes; they play a crucial role in bridging innate and acquired immunity in the liver [121]. However, hepatic and peripheral NK cells exhibit distinct immunophenotypic and functional properties. The functions of NK cells are tightly regulated by the stimulation of various excitatory and inhibitory surface receptors. Numerous studies have demonstrated that NK cells can be activated in NAFLD in response to increased levels of several NK-cell-activating cytokines and ligands [121,122]. However, conflicting reports suggest that the cytotoxic activity of NK cells is reduced in obese individuals with NAFLD and in mice fed a diet deficient in methionine and choline [122]. Therefore, additional investigations are necessary to elucidate the role of NK cells in NAFLD.

Natural killer T (NKT) cells are a unique subset of immune cells, expressing both specific NK cell surface receptors and antigenic receptors characteristic of conventional T cells. These cells are primarily located in the sinusoids, providing an intravascular immune surveillance [123]. Prior investigations have identified at least two NKT cell subsets with opposing roles in SI: type I NKT cells are pro-inflammatory, and type II provides protection against hepatic damage [124]. In mouse models, NKT-cell-deficient mice displayed increased susceptibility to the development of steatohepatitis and obesity when fed an HFD, while leptin-deficient mice showed reduced hepatic steatosis and improved glucose homeostasis upon the adoptive transfer of NKT cells [125,126]. NKT cell depletion is also associated with the activation of KCs and the production of local IL-12 [127]. In addition, a study reported that the severity of steatohepatitis is correlated with the enrichment of NKT cells in the liver [128,129]. Thus, NKT cells appear to be depleted during the development of hepatic steatosis but may increase later in the NAFLD process, contributing to fibrosis and inflammation [130].

## 4. Conclusions

NAFLD is gradually becoming a global pandemic. Unfortunately, limited options are available for the treatment of NAFLD. We summarized the substantial role of DAMPs, PRRs, and cellular interactions in the progression of NAFLD. Overall, the innate immune system has a significant impact on the pathogenesis of NAFLD, and targeting the innate immune system may be a promising approach for the development of new therapies for NAFLD. Further studies are needed to identify effective molecules that will be valuable in the treatment of NAFLD.

## Figures and Tables

**Table 1:** A list of DAMPs, PRRs, their functions, and release pathways for the development of NAFLD.

DAMP	PRR	Function	Release Pathway
Mitochondrial DNA	TLR9, NLRP3	Gene expression and NLRP3 inflammasome activation	Mitochondrial damage Plasma membrane permeabilization
Nuclear DNA	TLR9, NLRP3	Gene expression and NLRP3 inflammasome activation	Nuclear and plasma membrane permeabilization
HMGB1	TLR4	Multifunctional nuclear factor	Plasma membrane permeabilization
ATP	P2X7	Unclear	Plasma membrane permeabilization
Uric acid	NLRP3	Pro-inflammatory metabolite	Plasma membrane permeabilization

**Table 2:** Various immune cells and their ligands are involved in the development of NAFLD.

Cell	Function	Activating Ligand	Effector Ligand
Kupffer cell	▪ Removes cellular debris ▪ Macrophage chemotaxis	▪ DAMPs ▪ IL-1β ▪ C-X-C motif chemokine ligand	▪ TNF-α ▪ IL-6 ▪ TGF-β
Dendritic cell	▪ Tissue infiltration ▪ Controversial function in NAFLD	▪ X-C motif chemokine ligand	▪ IL-6 ▪ TNF-α ▪ MCP-1
Neutrophil	▪ Acute inflammatory response ▪ Phagocytosis	▪ IL-6 ▪ Hyaluronic acid	▪ Intercellular adhesion molecules ▪ IL-17A
NK cell	▪ Cytolytic activity	▪ IL-12 ▪ IL-15	▪ IFN-γ ▪ G-CSF

## Data Availability

Not applicable.
